# Maternal and Offspring Sugar Consumption Increases Perigonadal Adipose Tissue Hypertrophy and Negatively Affects the Testis Histological Organization in Adult Rats

**DOI:** 10.3389/fcell.2022.893099

**Published:** 2022-06-17

**Authors:** Gabriela Córdoba-Sosa, Leticia Nicolás-Toledo, Margarita Cervantes-Rodríguez, Nicté Xelhuantzi-Arreguin, María de Lourdes Arteaga-Castañeda, Elena Zambrano, Estela Cuevas-Romero, Jorge Rodríguez-Antolín

**Affiliations:** ^1^ Doctorado en Ciencias Biológicas, Universidad Autónoma de Tlaxcala, Tlaxcala, Mexico; ^2^ Centro Tlaxcala de Biología de la Conducta, Universidad Autónoma de Tlaxcala, Tlaxcala, Mexico; ^3^ Licenciatura en Nutrición, Facultad de Ciencias de la Salud, Universidad Autónoma de Tlaxcala, Tlaxcala, Mexico; ^4^ Licenciatura en Medicina, Universidad Popular del Estado de Tlaxcala, Tlaxcala, Mexico; ^5^ Licenciatura en Enfermería y Obstetricia, Facultad de Ciencias de la Salud, Universidad Autónoma de Tlaxcala, Tlaxcala, Mexico; ^6^ Departamento de Biología Reproductiva, Instituto Nacional de Ciencias Médicas y Nutrición Salvador Zubirán, Ciudad de México, Mexico

**Keywords:** maternal programming, perigonadal adipose tissue, hypertrophy, testis, high sugar intake

## Abstract

Sugar intake has been associated with the development of male reproductive pathologies because of the increase and dysfunction in different adipose tissue depots. The establishment of these dysfunctions in the early stages of development is unknown. We evaluated the effect of maternal (pregnancy and lactation) and male offspring (from weaning to adulthood) consumption of 5% sucrose on perigonadal adipose tissue (PAT) and testis in adulthood. Moreover, two rat groups were compared, both including pregnant and lactating females: Control (C—drinking tap water) and sugar (S—consuming 5% sucrose solution). From weaning to adulthood with male offspring, four subgroups were formed: Control Mother → Control and Sugar offspring (CC, CS) and Sugar Mother → Control and Sugar offspring (SC, SS). At 120 postnatal days, the testes and PAT were collected and morphologically described. Furthermore, we quantified the number and cross-sectional area of perigonadal adipocytes and their distribution. We found that the males from SC and SS groups showed high PAT weight (*p* < 0.005), a high number (*p* < 0.05), and a relative frequency of large adipocytes (*p* < 0.05), establishing these results during gestational and lactation stages, and enhancing in adulthood since postnatal diet and its interaction. More macrophages, mast cells, and Leydig cells were observed in the interstitial space of the testis for the CS, SC, and SS groups, concluding that consumption of a high-carbohydrate maternal diet, program hypertrophy processes in adult PAT, developing and enhancing with sugar consumption during postnatal life. Furthermore, they are associated with inflammatory processes within the interstitial space of the testis.

## Introduction

The caloric intake is closely related to diets rich in simple and rapidly assimilated sugars, such as glucose, fructose, and sucrose (Murphy and Johnson, 2003). Although the daily recommendation is 5%–10% of carbohydrates from the total energy intake in adults and children (Organization for Economic Co-operation and Development (OECD), 2017), the worldwide consumption of sugars has increased from 169 to almost 180 million metric tons (Murphy and Johnson, 2003; United States Department of Agriculture (USDA), 2020). This increase in the sugar consumption is present from gestation (Gamba et al., 2019; Casas et al., 2020) and breastfeeding (Zou et al., 2012), continuing throughout childhood (Dubois et al., 2007; De León-Ramírez et al., 2021) and adult life (Kumar et al., 2014). During gestation and breastfeeding, nutrition through the maternal diet plays a critical role (Cervantes-Rodríguez et al., 2014; Nicolás-Toledo et al., 2018; Pedrana et al., 2020), it is already well known that imbalances in the adequate consumption of macronutrients, such as proteins (Pedrana et al., 2020) and carbohydrates (Casas et al., 2020), negatively affect the development and maturation of different organs in later stages (Cervantes-Rodríguez et al., 2014; Nicolás-Toledo et al., 2018; Corona-Quintanilla et al., Online ahead of print) because both the glucose and fructose cross the placenta (Holmberg et al., 1956) and fetal development depends on transport of glucose through the mother’s blood (Regnault et al., 2013). These programmed or established changes during the fetal/embryonic stage that can cause diseases in adulthood are known as the theory of the origin and development of diseases (Barker, 1990).

Both high-calorie diets (Jastrzębska et al., 2014; De León-Ramírez et al., 2021) and a BMI > 25 kg/m^2^ (overweight and obesity) (Organization for Economic Co-operation and Development (OECD), 2017; Morales et al., 2014) is associated with male reproductive tract diseases related to some metabolic disorders (metabolic syndrome, insulin resistance, and dyslipidemias) (Nicolás-Toledo et al., 2018; De León-Ramírez et al., 2021). In animal models, high-carbohydrate diets have been established depending on the type and amount of mono, di, or polysaccharide used (Rodríguez-Correa et al., 2020). Also, it has already been described that consumption of these diets during pregnancy and postnatal stages can affect the function of the striated muscles associated with copulation (Corona-Quintanilla et al., Online ahead of print) and the expression of factors related to fat, such as the insulin receptor and the development of spermatogonias (Mao et al., 2018). In addition, postnatal consumption negatively affects testicular histology, injuring the intra and extra tubular epithelium (De León-Ramírez et al., 2021).

In mammals such as humans and rats, metabolic relationship between the testis and white adipose tissue (WAT) that surrounds it, better known as perigonadal adipose tissue (PAT), has not yet been clarified (Bjørndal et al., 2011; Luong et al., 2019) and even less the effect of high-carbohydrate diets on communication between both organs, especially if we consider that one of the main targets of metabolic pathologies associated with reproductive tract is adipose tissue, which is particularly vulnerable to changes in nutrition (Bibee et al., 2011).

Considering that limits of the perimeter of adipocytes observed under a histological slide resemble a polygonal mosaic whose vertices point outwards, like a Poisson–Voronoi diagram, the gamma distribution has been used as a statistical model proposal to characterize the adipocyte size distribution (Ibáñez et al., 2018). In this report, measures associated with the spread and shape of the data distribution under the assumption of gamma distribution are used to characterize the size distribution of perigonadal adipocytes and to determine, based on the theory of the developmental origins of the health and disease, its causal relationship with maternal consumption of sucrose and postweaning in male offspring.

The development of alterations in the male reproductive tract has been associated with sugar consumption and metabolic disorders such as for overweight and obesity (Martini et al., 2010; Sadeghi-Bazargani et al., 2013). The progress of these diseases is characterized by an abnormal or excessive accumulation of lipids in the adipocytes of different deposits of WAT (Cinti et al., 2019), as a result of the consumption of high-calorie diets (França et al., 2020; Oliveira et al., 2020). The PAT is one of its largest fat deposits (Berry et al., 2016) and it is the most important for the testis since its extraction in one or both gonads inhibits spermatogenesis (Chu et al., 2010). Although the joint consumption of carbohydrates and fats during pregnancy and postnatal life has adverse effects on the development of germ, Sertoli, and Leydig cells in mice (Mao et al., 2018), and the effect on PAT and testicular epithelium specifically by chronic consumption of simple sugars through maternal diet and throughout postnatal life is still unknown. For this, our objective was to determine the effect of 5% sucrose consumption in maternal (gestation and breastfeeding) and postnatal (childhood and adulthood) stages on the testis and PAT in adult male rats. We hypothesize that maternal consumption of sucrose may program the development of PAT hypertrophy, as well as histological alterations in the testes of male offspring; and this programming could be maintained and enhanced with the consumption of the same disaccharide from early childhood (weaning) to adulthood.

## Materials and Methods

### Animals

All procedures applied to animals were approved by the Bioethics Committee the Centro Tlaxcala de Biología de la Conducta from Universidad Autónoma de Tlaxcala, following the Mexican guide for animal care (NOM-062-Z00-1999, Mexico). Twelve pregnant female (14-week-old) Wistar rats (*Rattus norvegicus*), weighing 220–240 g, were housed individually in polypropylene cages (37 cm × 27 cm × 16 cm) and maintained in controlled rooms with temperatures of 23 ± 1°C and a 12-h light–dark cycle (with the light off from 08:00 h to 20:00 h).

### Experimental Design

**FIGURE 1:**
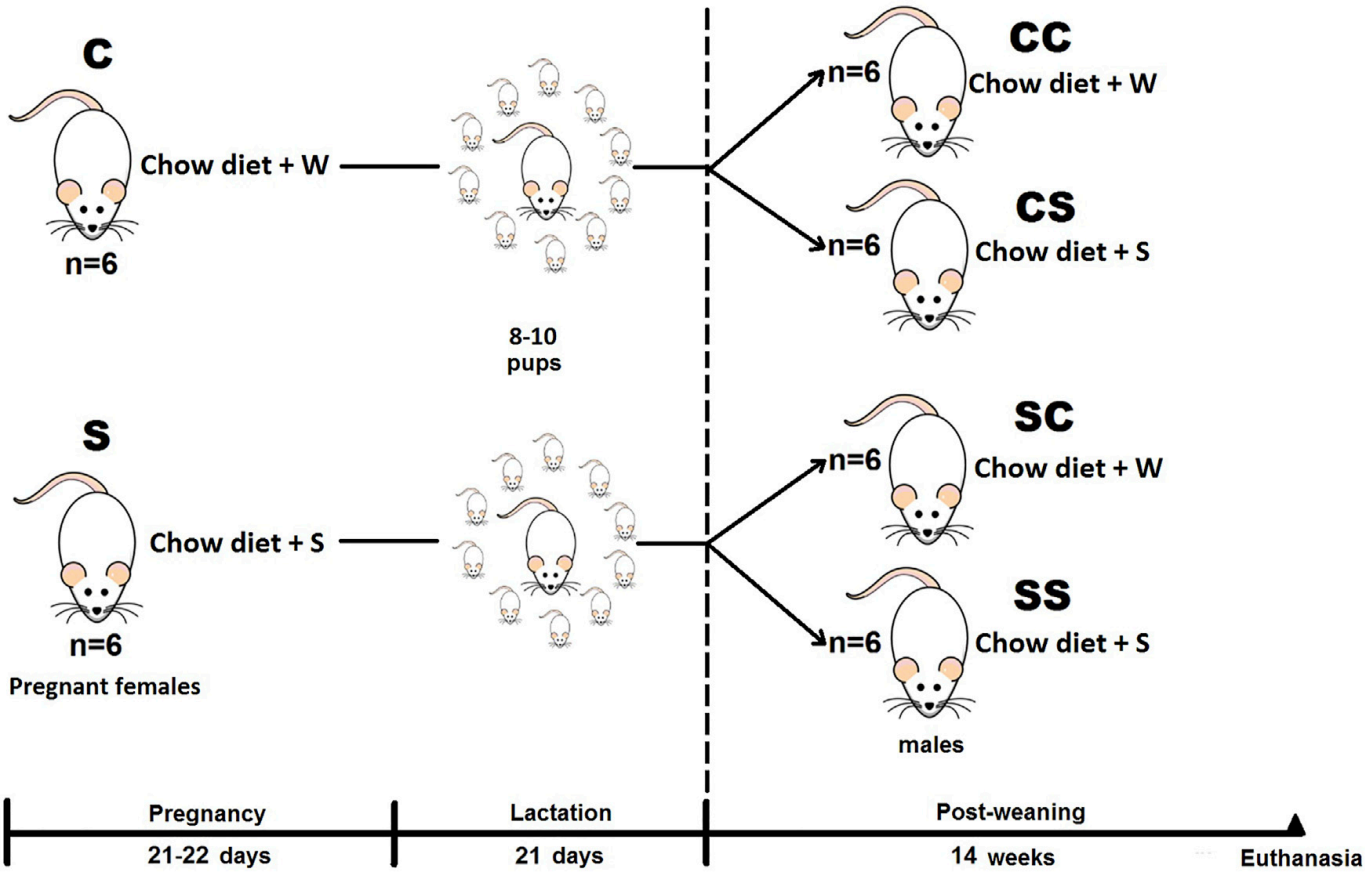
Experimental design. Groups of pregnant and breastfeeding females, and from male rats from weaning to adulthood, fed with a standard diet and tap water (W) without (C) and added 5% sucrose (S).

The details of the experimental design used in this study have been published previously (Corona-Quintanilla et al., Online ahead of print). Primiparous female rats were paired with males of proven fertility. The female was individually placed in polypropylene boxes, considering the day of mating as day 0 of gestation. During pregnancy and breastfeeding, the females were randomly assigned into two groups: a control group (C), which drank unadulterated tap water, and an experimental group that was provided a 5% sucrose solution (S) to drink. Postpartum, the litters were adjusted to 8–10 pups and from postnatal day 22–120, and the male offspring were in pairs or trios placed in polypropylene boxes and fed with either tap water or 5% sucrose according to the assigned experimental group: control mother-control offspring (CC), control mother-sucrose offspring (CS), sucrose mother-control offspring (SC), and sucrose mother-sucrose offspring (SS) (Figure 1).

### Dietary Protocol

All rats were fed daily with Chow-5001 standard diet (LabDiet 5001-Laboratory Rodent Diet, St. Louis, MO, United States) and tap water *ad libitum*, and 5% sucrose was diluted in the tap water for the S groups. The conditions of maintenance and care of the animals were previously established (Corona-Quintanilla et al., Online ahead of print). Notably, this amount of sugar added to the diet does not decrease the energy intake of the equivalent to 15% protein, as we have previously reported (Cervantes-Rodríguez et al., 2014; Corona-Quintanilla et al., Online ahead of print), because the consumption of this nutrient can be considered deficient when it is very low (3% and 5%) and moderately low (8% and 12%), or adequate when equal to 15% and 20% (Moro et al., 2021). This study aims to investigate the effect of sugar consumption without the interference of protein deficiency.

Water and food consumption and body weight were measured daily at 9:30 h during pregnancy, breastfeeding, and adulthood (offspring) (Corona-Quintanilla et al., Online ahead of print) to calculate weight gain, consumption of carbohydrates, lipids, and proteins, as well as their respective individual and total caloric intake. These parameters were daily evaluated according to the nutritional description of the Chow 5001 food.

### Obtaining Tissues

Before euthanasia, males from 120 postnatal days (PNd) fasted for 12 h. Male rats were deeply over-anesthetized with sodium pentobarbital (50 mg/kg BW) and decapitated using a rodent guillotine device (Harvard Apparatus, Holliston, MA). After decapitation, animals were placed in a supine position and made a longitudinal incision on the abdominal cavity. Both testes with the epididymis and PAT were harvested and weighed (g tissue/100 g BW) (De León-Ramírez et al., 2021).

### Morphometric Analysis

At birth, pup weight, body length (from the tip of the nose to base of tail), heat diameter, abdominal diameter, and anogenital distance were measured using calipers. Left testes were removed, fixed, and dehydrated according to the procedure established by De León-Ramírez et al. (2021). They were embedded in Paraplast Plus (Sigma-Aldrich. St. Lois MO, United States), cut at 7 μm, and stained with hematoxylin and eosin (H&E) (De León-Ramírez et al., 2021). Sections were visualized and photographed using a light microscope (Zeiss Axio Imager A1) at ×2.5, ×4, ×10, and ×40. For the cross-sectional area (CSA) and number of transversally cut seminiferous tubules quantification, reconstructions of microphotographs were conducted at ×4 using the Adobe Illustrator CS5 program. The analysis of histological characteristics of the seminiferous epithelium was performed according to Morales et al. (2014). After excision, random samples of left gonadal fat were fixed, dehydrated, cut, and stained according to the protocol of Cervantes-Rodríguez et al. (2014). Slides were collected forming a series of 10 sections and discarding 10 until 30 sections were obtained per sample. Adipocytes were observed using an optical microscope (Axio Imager A1, Zeiss) coupled to an Olympus digital camera.

The number and CSA of adipocytes were measured with the support of a grid in the central zone of the photomicrographs obtained at ×10 and ×40 during the reconstruction of the PAT sections photographed with Axiovision 4.8 software (Carl Zeiss MicroImaging, Inc.). Relative frequency histograms were plotted with 200 μm^2^ CSA intervals (i.e., 0–200, 201–400, and 401–600 μm^2^) up to the maximum observed CSA interval.

### Estimation of Small and Large Adipocyte Proportions

The cutoff points for small, medium, and large adipocytes were defined based on the 25th and 75th percentiles of the relative distribution of the CC group and analysis by non-parametric comparisons.

### Statistical Analyses

The sample size in each group was six rats from different litters. Following the normality of the data (with a frequency distribution histogram), the comparison of groups of mothers during pregnancy and breastfeeding was done by Student’s t test.

Two-way ANOVA was used to compare data of body weight, weight gain, food intake, water consumption, testicular, and PAT parameters (number, weight, and CSA of seminiferous tubules and adipocytes, as well as adipocytes size—small, medium, and large proportion) in male offspring for all groups by the combined effects of period diet and sucrose consumption. Period diet (maternal consumption: gestation/breastfeeding; postnatal: childhood/puberty/adulthood) was considered as the first independent variable and sugar intake as the second independent variable. Where ANOVA indicated a significant (*p* < 0.05) effect of treatments, a *post hoc* test was carried out using the Tuckey correction to determine significant differences.

The CSA distribution of adipocytes and seminiferous tubules was analyzed using frequency histograms and subsequently Fisher’s test by *X* ^2^ was used to compare the percentage of area according to size bin.

GraphPad Prism Version 6.0 program for Windows was used and all variables measured were expressed as means ± SEM. Significant differences were considered with a p ≤ 0.05 for all cases.

## Results

### Growth and Nutritional Consumption of Pregnant Mothers

Differences in weight and consumption of water and food are shown in the Table 1. During pregnancy, there were no differences between the C and S groups in the body weight at beginning of gestation nor in weight gain, but 5% sucrose intake affected the weight, showed at the end of pregnancy (Table 1). Throughout breastfeeding, there were no differences in initial, final, or weight gain.

**TABLE 1:** Growth and metabolic parameters of adult females that consumed simple (C) and 5% sucrose (S) water during gestation and breastfeeding.

	Pregnant females			
	C	S	Variation (%)	p values
Pregnancy initial body weight (1 GD) (g/d/100 g BW)	232 ± 3.1	240 ± 3.5	3.4^a^	0.1235
Pregnancy final weight (21 GD) (g/d/100 g BW)	366 ± 2.0	382 ± 6.3	4.4^a^	0.0342
Pregnancy weight gain (1–21 GD) (g/d/100 g BW)	134 ± 3.2	142 ± 6.2	6.2^a^	0.2613
Breastfeeding initial body weight (1 PNd) (g/d/100 g BW)	274 ± 6.5	270 ± 7.3	1.6^b^	0.6506
Breastfeeding final body weight (21 PNd) (g/d/100 g BW)	308 ± 10.4	300 ± 5.5	3.6^b^	0.5150
Breastfeeding weight gain (1–21 PNd) (g/d/100 g BW)	38.8 ± 2.5	34.7 ± 4.9	10.6^b^	0.4728
Food consumption (g/d/100 g BW)	8.7 ± 0.1	6.7 ± 0.3	23.1^b^	0.0002
Water consumption (ml/d/100 g BW)	21.6 ± 1.2	42.8 ± 6.7	98.0^a^	0.0109
Carbohydrate consumption (g/100 g BW)	88.1 ± 2.7	108.6 ± 4.8	23.2^a^	0.0045
Carbohydrate energy intake (kcal/100 g BW)	352 ± 11.1	434 ± 19.5	23.2^a^	0.0045
Lipid consumption (g/100 g BW)	18.7 ± 0.3	14.4 ± 0.6	23.1^b^	0.0002
Lipid energy intake (kcal/100 g BW)	169 ± 3.3	130 ± 6.1	23.0^b^	0.0002
Protein consumption (g/100 g BW)	41.9 ± 0.8	32.2 ± 1.5	23.0^b^	0.0002
Protein energy intake (kcal/100 g BW	167 ± 3.3	129 ± 6.1	23.1^b^	0.0002
Total energy intake (kcal/100 g BW)	689 ± 14.9	693 ± 18.9	0.6^a^	0.8613

The mean values ± SEM (*n* = 6) are observed.

a Increase in the percentage variation of metabolic parameters respect control group.

b Decrease in the percentage variation of metabolic parameters respect control group.

GD, gestational day; PNd, postnatal day, BW, body weight.

During maternal consumption period, females from the S group significantly increased their intake of both sugar water (98%) and total carbohydrates (by ∼ 23%). Likewise the consumption of solid food (lipids and proteins) decreased ∼ 23%; (Table 1). Respect to the protein consumption, it is important to mention that the caloric intake in the S group of dams was equivalent to 18.5%, while 24.1% was for the control group.

### Morphometric Measurements of Pups at Birth

Male offspring from dams who consumed sucrose during pregnancy and breastfeeding did not show effects on their initial or final breastfeeding weight, length, head, and abdominal circumference, or anogenital distance (Table 2).

**TABLE 2:** Weight, morphometric measurements, and water and food intake obtained at the birth, breastfeeding (from 1 to 21 PNd), and postnatal stage (22 to 120 PNd) of male offspring that consumed sucrose at 5% (CS and SS) and simple (CC and SC) water and whose mothers during gestation and breastfeeding consumed the same diets. The effect of diets during the pre (MD) and postnatal (PDN) periods and their interaction are shown.

	Pups		
	C	S	p-value
Weight 1 PNd (g)	7.1 ± 0.3	7.5 ± 0.3	0.3819
Weight 21 PNd (g)	38.2 ± 2.5	39.0 ± 1.5	0.7929
Weight gain 1 to 21 PNd (g)	31.0 ± 2.4	31.5 ± 1.7	0.8787
Length (mm)	49.4 ± 0.8	51.9 ± 0.9	0.8027
Head circumference (mm)	12.0 ± 0.3	11.7 ± 0.2	0.5083
Abdominal circumference (mm)	14.8 ± 0.9	15.4 ± 0.4	0.5440
Anogenital distance (mm)	3.4 ± 0.1	3.4 ± 0.1	0.9071

	Offspring male groups				p-value		
	CC	CS	SC	SS	MD	PND	MD × PND interaction
Average initial feed (21–28 PNd) intake (g/d/100 g BW)	17.3 ± 0.8	15.1 ± 0.2	17.0 ± 1.4	14.0 ± 0.5	0.3988	0.0085	0.6484
Average final feed (113–120 PNd) intake (g/d/100 g BW)	6.5^a^ ± 0.3	4.4^b^ ± 0.2	5.9^a^ ± 0.4	4.3^b^ ± 0.4	0.3824	˂ 0.0001	0.4984
Average total feed intake (g/d/100 g BW)	9.4^a^ ± 0.3	7.6^bc^ ± 0.1	9.0^ac^ ± 0.4	7.5^b^ ± 0.4	0.4539	0.0002	0.6058
Initial water (21–28 PNd) intake (ml/d/100 g BW)	37.6 ± 3.4	46.2 ± 5.0	34.1 ± 4.7	38.7 ± 2.7	0.1968	0.1243	0.6238
Final water (113–120 PNd) intake (ml/d/100 g BW)	15.0^a^ ± 1.2	30.5^b^ ± 2.8	13.1^a^ ± 1.9	29.4^b^ ± 4.1	0.6060	˂ 0.0001	0.8882
Total water intake (ml/d/100 g BW)	19.7^a^ ± 1.2	31.5^b^ ± 1.8	19.2^a^ ± 2.4	30.2^b^ ± 2.5	0.6564	˂ 0.0001	0.1079

Mean values ± SEM (*n* = 6) are observed. Superscript letters indicating differences between groups. Data analyzed with a two-way ANOVA and Tukey *post hoc*. MD: Maternal diet, PND: postnatal diet, PNd: Postnatal day, BW: body weight.

### Growth and Nutritional Consumption of Males During Postnatal Stage

**FIGURE 2:**
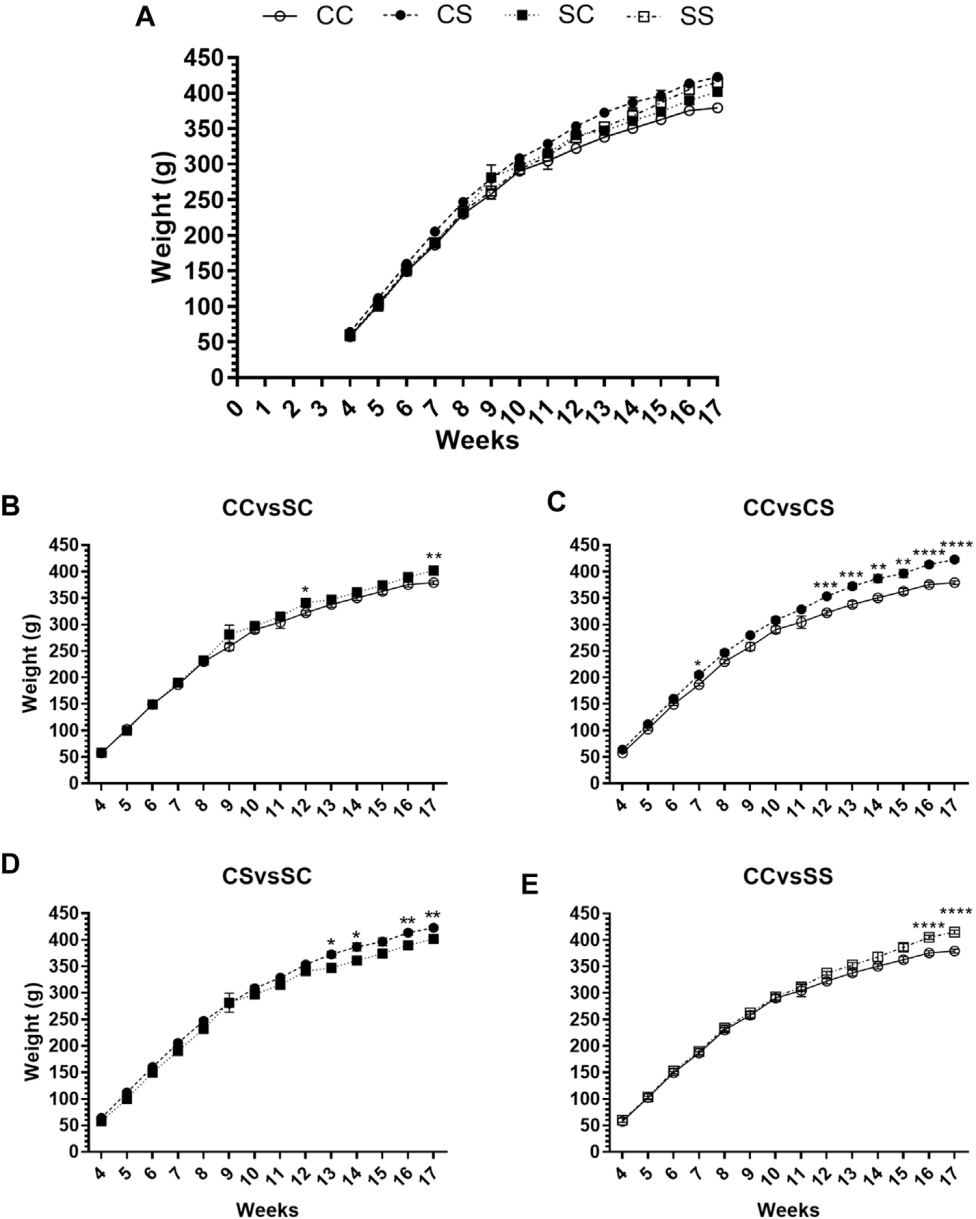
Weekly weight gain **(A)** of CC (white circle and solid line), CS (black circle with dotted line), SC (black square with dotted line), and SS (white square with dotted line) male groups from weaning at 120 PNd. Comparisons per week between groups of males that presented differences due to maternal **(B)** or postnatal diet **(C)** and interaction **(D,E)**. Mean values ±SEM (*n* = 6) are shown. Data were analyzed with a two-way ANOVA and Tukey *post hoc*. *p < 0.05, **p < 0.01, ***p < 0.001, and ****p < 0.0001.

The sucrose intake did not modify the body weight (g) at 22 PNd (CC: 57 ± 2.2, CS: 64 ± 2.8, SC: 58 ± 3.2, and SS: 60 ± 4.1) at any life period (maternal consumption diet: *p* = 0.5732; postnatal diet: *p* = 0.1960; and interaction: *p* = 0.4807). There was an increase in the weight gain after 10 weeks in those groups that consumed sucrose at some period of their development, compared to the CC group (Figure 2A). This increase in the weight varied accordingly to the period of life in which sucrose was intake. Significant differences were found from 12 to 17 weeks since prenatal intake (CC vs. SC, Figure 2B), during the 7th week and from 12 to 17 weeks since postnatal consumption (CC vs. CS, Figure 2C), and from 13 to 17 weeks since consumption interaction in both periods (CS vs. SC and CC vs. SS; Figures 2D,E).

At 120 PNd, the weight (g) increased with sugar intake (CC: 379 ± 3.6, CS: 422 ± 3.7, SC: 401 ± 3.7, and SS: 415 ± 3.6) exhibiting significant differences between CC vs. CS (*p* ≤ 0.0001), CC vs. SC (*p* ≤ 0.005), CC vs. SS (*p* ≤ 0.0001), and CS vs. SC (*p* ≤ 0.005). A weight gain (g) was also observed from 22 to 120 PNd (CC: 321 ± 3.7, CS: 358 ± 5.3, SC: 343 ± 4.0, and SS: 355 ± 6.0), with significant differences between the groups CC vs. CS (*p* ≤ 0.05), CC vs. SC (*p* ≤ 0.05), and CC vs. SS (*p* ≤ 0.005).

The postnatal sucrose intake had effect on both the body weight at 120 PNd (*p* ≤ 0.0001) and weight gain (*p* ≤ 0.0001) (Figures 2A,C). However, there was a significant effect by the interaction (Figures 2D,E) of maternal and postnatal diets (final weight: *p* = 0.0005 and weight gain: *p* = 0.0160). The effect of the postnatal diet was enhanced by the maternal diet (final weight, *p* = 0.0596 and weight gain, *p* = 0.0740).

All data related to water and food intake in male groups are presented in the Table 2.

The initial, final, and total food consumption at the postnatal period was significantly decreased only in the groups with 5% sucrose postweaning intake. At the beginning of this study, there were no differences in the food intake between the groups. However, at the final of treatment, the food intake (CC vs. CS *p* ≤ 0.005; CC vs. SS *p* ≤ 0.005; CS vs. SC *p* ≤ 0.05; and SC vs. SS *p* ≤ 0.05) and total consumption (CC vs. CS *p* ≤ 0.005; CC vs. SC *p* ≤ 0.005; and SC vs. SS *p* ≤ 0.05) were different.

The initial water consumption was not affected by the maternal or postnatal sucrose intake, nor by the interaction (Table 2). However, in the postnatal stage, the final (CC vs. CS *p* ≤ 0.005; CC vs. SS *p* ≤ 0.005; CS vs. SC *p* ≤ 0.005; and SC vs. SS *p* ≤ 0.005) and total (CC vs. CS *p* ≤ 0.005; CC vs. SS *p* ≤ 0.005; CS vs. SC *p* ≤ 0.005; and SC vs. SS *p* ≤ 0.005) water consumption was significantly increased by 5% sucrose intake.

Data related to nutritional intake are presented in the Table 3. The decrease in the consumption of both proteins and lipids and their respective energy calculation in the CS and SS groups were due to consumed postnatal sucrose with extremely significant effect (corresponding to 49% of the variance in said variables), as well as significant differences were observed between groups. In the CS, SC, and SS groups, the decrease in the lipids and proteins was 19%, 4.5%, and 20%, respectively. Specifically, the equivalent of protein caloric intake in these groups ranged between 19% and 23% (CS: 19.5%; SC: 23.0%; and SS: 19.2%), compared to the CC group which was 24.1%.

**TABLE 3:** Sum of total consumption and energy intake of proteins, lipids, and carbohydrates from 4 to 17 weeks, and parameters testis in the groups of males that consumed sucrose at 5% (CS and SS) and simple (CC and SC) water during the postnatal stage (22 to 120 PNd) and whose mothers during pregnancy were fed the same diets. The effect of diets during the maternal (MD) and postnatal (PND) periods and their interaction are shown.

	Offspring male groups				p-value		
	CC	CS	SC	SS	MD	PND	MD × PND interaction
Total protein intake (g/d/100 g BW)	31.5^a^ ± 1.0	25.4^bc^ ± 0.4	30.1^ac^ ± 1.6	25.1^b^ ± 1.4	0.4840	0.0002	0.6446
Protein energy intake (kcal/d/100 g BW)	126.1^a^ ± 4.0	101.9^bc^ ± 1.7	120.4^ac^ ± 6.5	100.7^b^ ± 5.6	0.4837	0.0002	0.6453
Lipids intake (g/d/100 g BW)	14.1^a^ ± 0.4	11.4^bc^ ± 0.2	13.4^ac^ ± 0.7	11.2^b^ ± 0.6	0.4842	0.0002	0.6433
Lipids energy intake (kcal/d/100 g BW)	127.0^a^ ± 4.0	102.6^bc^ ± 1.8	121.2^ac^ ± 6.5	101.4^b^ ± 5.7	0.4836	0.0002	0.6448
Carbohydrates intake (g/d/100 g BW)	64.2^a^ ± 2.0	73.9^ab^ ± 1.0	61.3^ac^ ± 3.3	72.4^a^ ± 4.1	0.4515	0.0017	0.8078
Carbohydrates energy intake (kcal/d/100 g BW)	257^a^ ± 8.1	295^ab^ ± 4.0	245^ac^ ± 13.3	289^a^ ± 16.4	0.4519	0.0017	0.8075
Total energy intake (kcal/100 g BW)	510 ± 16.2	500 ± 5.6	487 ± 26.4	492 ± 27.3	0.4582	0.9076	0.7267
Hematocrit 120 PNd (%)	68.5 ± 1.7	66.3 ± 1.5	69.1 ± 0.5	62.1 ± 8.2	0.6843	0.2997	0.5780
Relative weight of testes (g)	0.48 ± 0.01	0.44 ± 0.01	0.47 ± 0.03	0.48 ± 0.02	0.6355	0.6143	0.3634
Number of sections counted of seminiferous tubules	468 ± 60.6	553 ± 51.4	489 ± 38.0	562 ± 56.6	0.7740	0.1416	0.9102
Seminiferous tubule cross-sectional area (CSA) (µm^2^)	52.1 ± 3.3	45.9 ± 1.6	51.3 ± 2.8	47.1 ± 2.0	0.9548	0.0550	0.7061

Mean values ±SEM (*n* = 6) are observed. Superscript letters indicating differences between groups. Data analyzed with a two-way ANOVA and Tukey *post hoc*. MD, Maternal diet, PND, postnatal diet, BW, body weight.

The increase in the carbohydrate consumption was 15% and 12% in the CS and SS groups compared to the CC group. This increase (38.7% of the total variance) was associated with postnatal carbohydrate consumption and its energy calculation. A significant difference between groups was found only between the CS and SC groups. Both the total caloric intake and hematocrit level in adult males for all groups were unaffected by maternal and postnatal diets, or their interaction.

### Morphometric Analysis of the Testis

**FIGURE 3:**
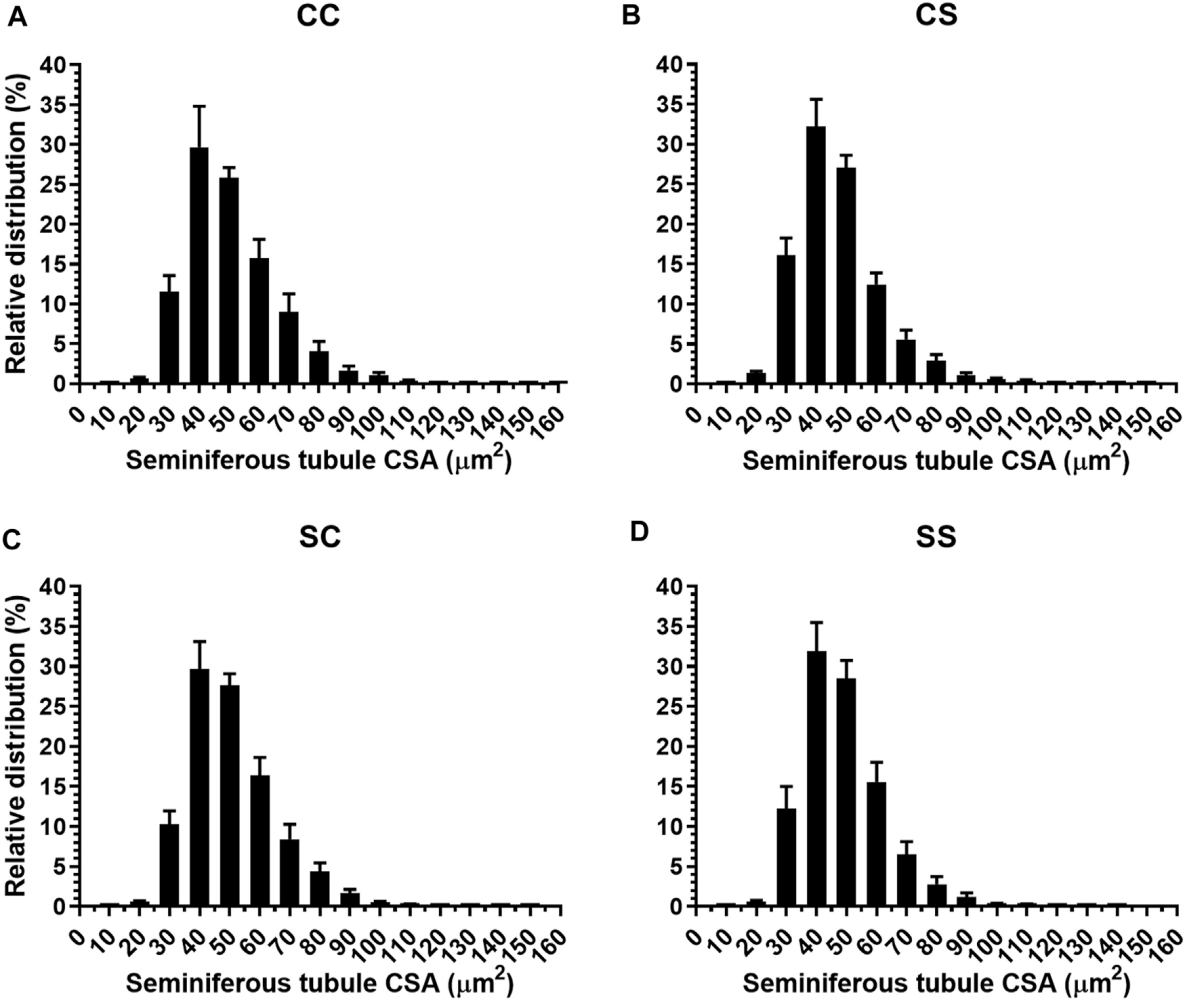
Percentage of the relative distribution of cross-sectional area (CSA) of the seminiferous tubules for the CC **(A)**, CS **(B)**, SC **(C)**, and SS **(D)** groups, who consumed or not 5% sucrose during the postnatal stage and whose mothers during gestation and breastfeeding were fed with same diets.

Testis variables such as the relative weight, CSA, and number of seminiferous tubules were unaffected by maternal and postnatal diets or their interaction (Table 3). Relative distribution of the CSA of the seminiferous tubules was carried out (Figure 3). The seminiferous tubules had CSA ranging from 20 to 120 μm^2^, finding the highest percentage (more than 50%) among 40–59 μm^2^, and presenting a Gaussian distribution around this interval. However, there were no differences between them (Figure 3).

**FIGURE 4:**
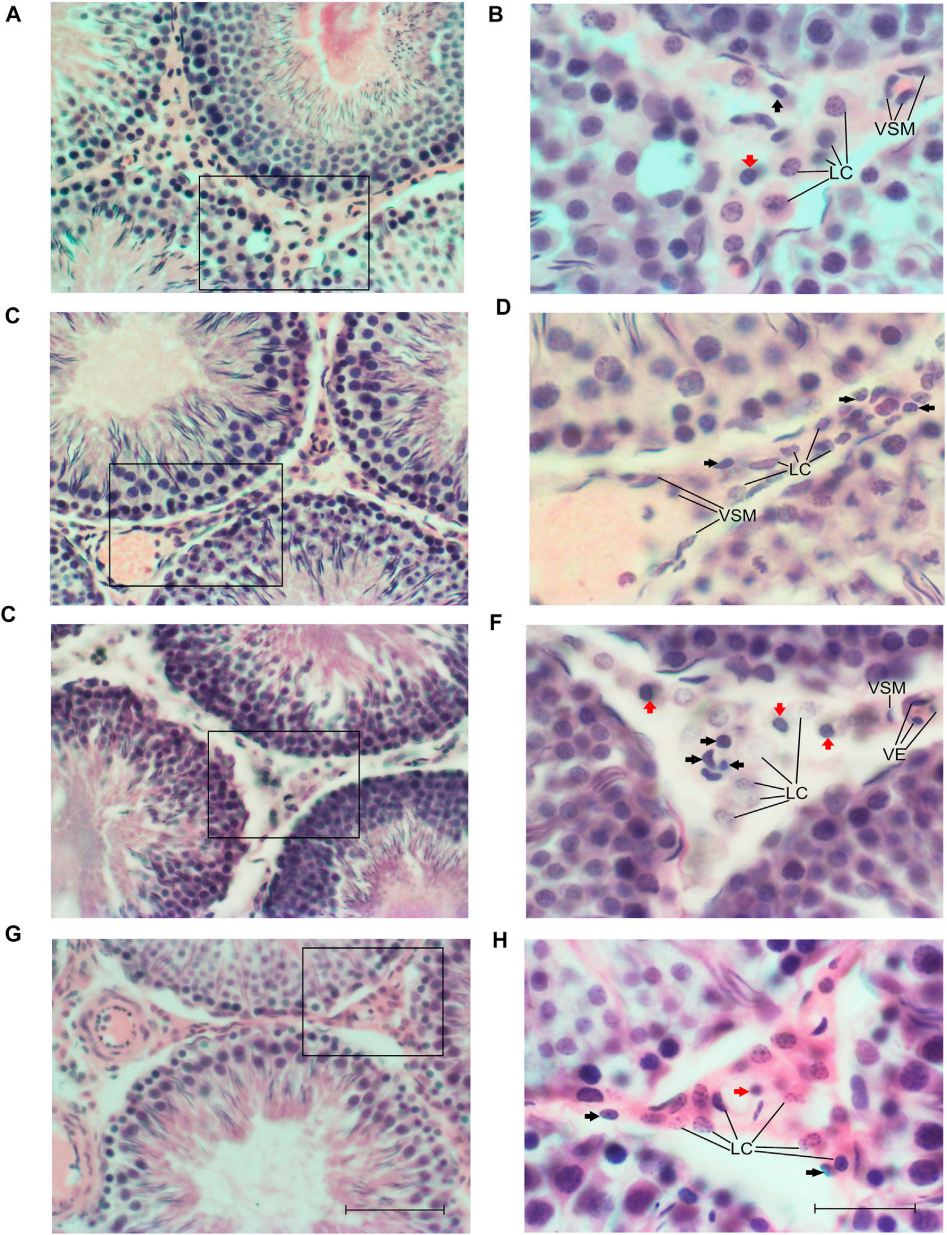
Photomicrographs of the cross-sectional area (CSA) of testis stained with H&E at ×10 (scale bar = 100 µm) (a, c, e, and g) and ×40 (scale bar = 50 µm) **(B, D, F, H)** for the CC **(A,B)**, CS **(C,D)**, SC **(E,F)**, and SS **(G,H)** groups. Abbreviations: Interstitial space of the Leydig cells (LC), blood vessels (BV), vascular epithelium (EV), vascular smooth muscle (VSM) cells, macrophages (black arrows), and mast cells (red arrows).

No differences in the cell arrangement, typical presence of Sertoli cells in the basal area, germ cells (spermatogonia, spermatocytes, spermatids, and sperm), tunica propria (peritubular tissue of myoid cells) of each tubule, Leydig cells, and other populations of interstitial cells were found between groups (Figures 4A–H). However, a high number of interstitial cells, macrophages, and mast cells were observed in those groups that consumed sucrose at some stage of their development (Figures 4A–H).

### Morphometric Analysis of Perigonadal Adipose Tissue

**FIGURE 5:**
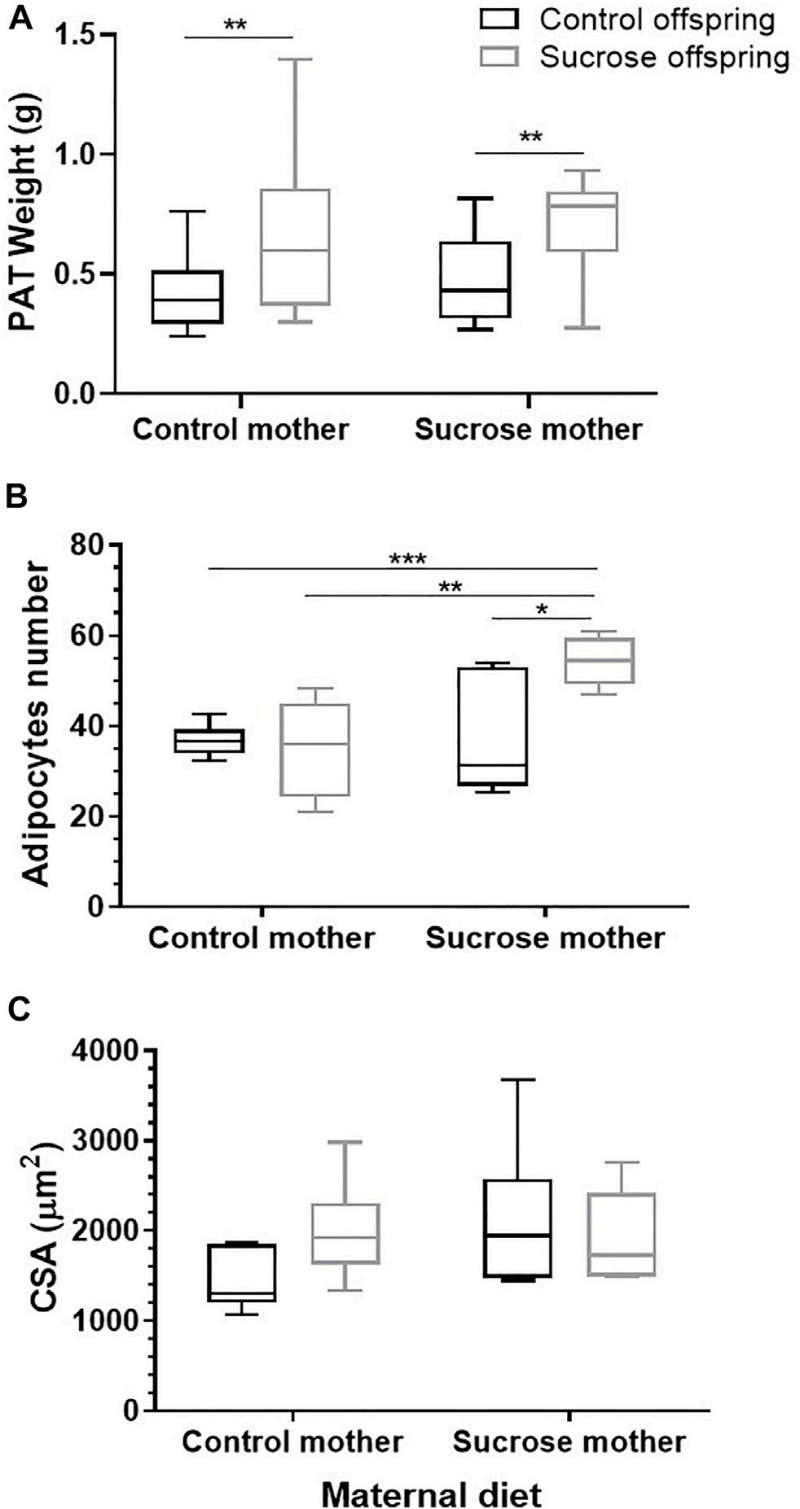
Relative weight of TAP **(A)**, number **(B)**, and cross-sectional area (CSA) **(C)** of adipocytes of males that consumed 5% sucrose (gray box) and tap water (black box) during the postnatal period and whose mothers during pregnancy and breastfeeding were fed with same diets. Data were analyzed with a two-way ANOVA and Tukey *post hoc*. Significant differences are shown. *p < 0.05, **p < 0.01, and ***p < 0.001.

The relative weight of the PAT (Figure 5A) for the CS (0.6 g ± 0.1) and SS (0.7 g ± 0.1) groups was significantly increased in comparison with the CC (0.4 g ± 0.1) and SC (0.4 g ± 0.1) groups, having 20.2% of variation to the effect of 5% sucrose postnatal consumption (*p* = 0.0044). Neither the maternal diet (*p* = 0.604) nor the interaction (*p* = 0.8774) of diets between both periods had any significant effect on this variable. The number of adipocytes (Figure 5B) was significantly increased by the maternal diet (*p* = 0.0189), interaction (*p* = 0.0214), and postnatal diet (*p =* 0.0528). *Post hoc* tests showed significant differences between the SS (54 ± 2.4) and the CC (36 ± 1.4), and the CS (35 ± 4.7) and SC (37 ± 5.2) groups.

For the CSA average (µm) of adipocytes (Figure 5C), no differences were found between the CC group (1439 ± 138) and those who consumed sucrose in the maternal (SC: 2117 ± 336) and/or postnatal (CS: 1996 ± 225; SS:1917 ± 215) periods [maternal (*p* = 0.2252) and postnatal (*p* = 0.4649) diets or by their interaction (*p* = 0.1291)].

### Frequency Distribution of Perigonadal Adipocytes

**FIGURE 6:**
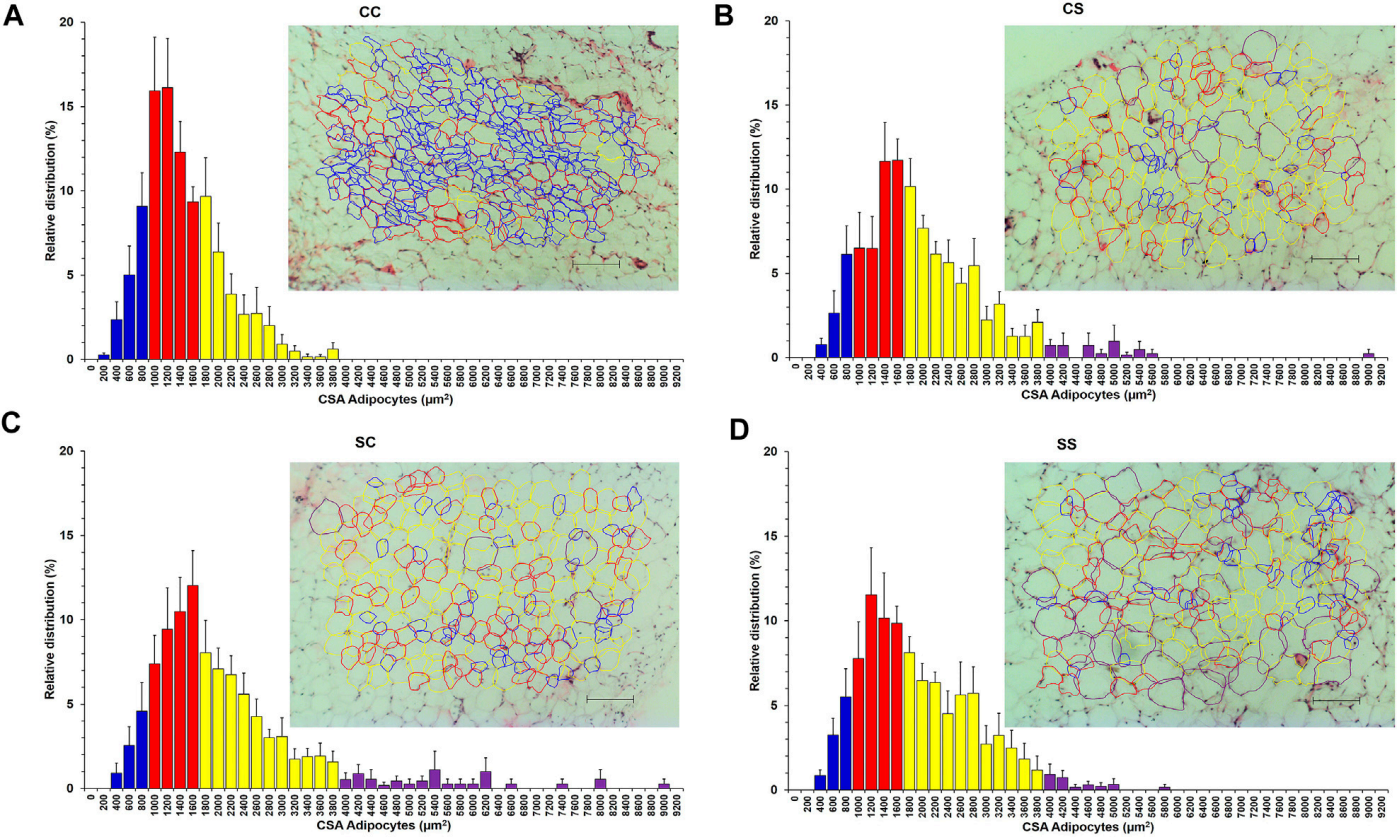
Relative frequency distribution of adipocytes counted according to cross-sectional area (CSA) for the CC **(A)**, CS **(B)**, SC **(C)**, and SS **(D)** groups. Mean values ±SEM (*n* = 6) are found. Photomicrographs of perigonadal adipocytes of CC **(A)**, CS **(B)**, SC **(C)**, and SS **(D)** groups stained with H&E at ×10 (scale bar = 100 µm). The color of bars in graphics corresponds with the outline drawn of adipocytes. Blue (small, ≤1,051 μm^2^), red (medium, from 1,052 to 1,788 μm^2^), yellow (large, ≥1,789 μm^2^), and purple (those with a CSA greater than 4,000 μm^2^).

The relative frequency distribution of adipocytes with intervals of 200 units in the four groups of males (Figure 6A) presented a Gaussian-type distribution, loaded toward the extreme left, with a high proportion of adipocytes with CSA smaller than 1051 μm^2^.

Only groups that consumed sucrose at some period of their life presented adipocyte proportions with CSA greater than 3,600 μm^2^ (Figures 6B–D), especially the SC and SS groups (Figures 6C,D), whose proportion of adipocytes with CSA of 4,800–9,000 μm^2^ was constant in almost all established intervals.

**FIGURE 7:**
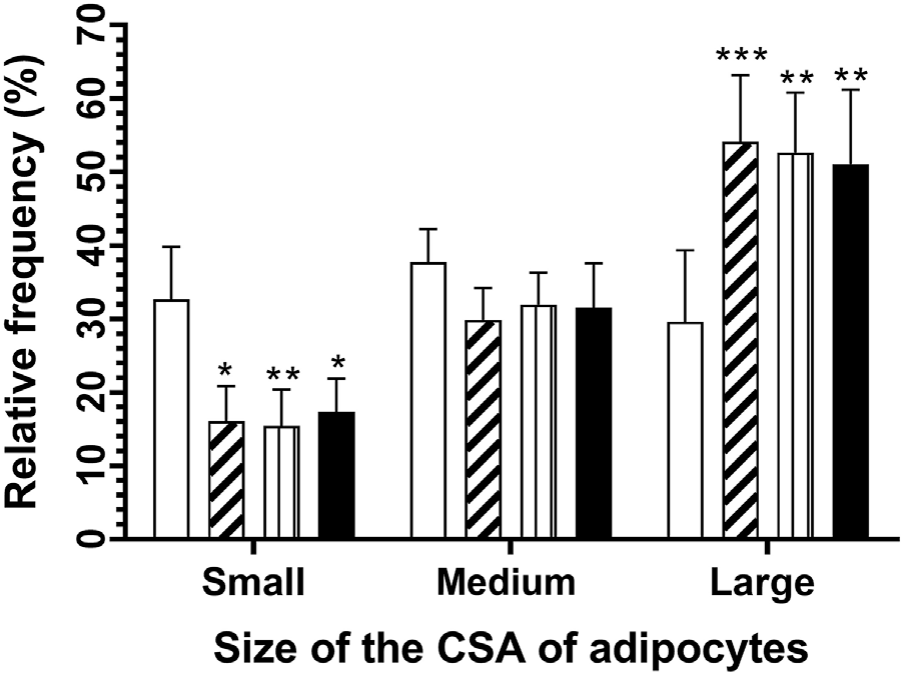
Relative frequency of CSA adipocytes according to their size category for the CC (white bars), CS (diagonal lines bars), SC (vertical lines bars), and SS (black bars) groups. Means ± SEM. Data were analyzed with a two-way ANOVA and Tukey *post hoc*. Significant differences among intervals are shown. *p < 0.05, **p < 0.01, and ***p < 0.001.

Comparisons were done between intervals of groups and showed that postnatal sucrose consumption decreased the proportion of adipocytes considered small (≤1,051 μm^2^), increasing proportions for large adipocytes (≥1,789 μm^2^) (Figure 7), being approximately 6% of adipocytes had CSA from 3,600 to 9,000 μm^2^ (Figures 6C,D).

Comparisons with Fisher’s test by *X* ^2^ between groups with intervals of 200 units were done, resulting in significant differences in adipocytes with CSA from 1001 to 1200 and 1201 to 1400 by postnatal diet (CC vs. CS, *p* = 0.0400). However, to properly distinguish the effect of diet in the two periods, one more comparison (data not plotted) was done with frequency intervals of 400 units. Resulting in a significant decrease in the proportion of CSA adipocytes from 801 to 1200 μm^2^ (*p =* 0.0281) by maternal diet (CC vs. SC) and in those from 1,201 to 1,600 μm^2^ (*p =* 0.0414) by postnatal diet (CC vs. CS). To corroborate these results, two comparisons were done with frequency intervals of 600 and 1200 μm^2^ (histograms are not shown), which found the same pattern of differences in the range from 1,201 to 1,800 μm^2^ (maternal diet, CC vs. SC: *p =* 0.0178; postnatal diet, CC vs. CS: *p =* 0.0072) and from 1,201 to 2,400 μm^2^ (maternal diet, CC vs. SC: *p =* 0.0005, postnatal diet, CC vs. CS: *p =* 0.0022).

### Estimation of Small and Large Perigonadal Adipocyte Proportions

The cutoff points for small and large adipocytes were defined based on the 25th and 75th percentiles of the CSA from CC group distribution (Figure 6A), which were 1,051 μm^2^ for small adipocytes and 1,789 μm^2^ for large ones.

When the relative percentage of small, medium, and large adipocytes between groups was compared (Figure 7), only significant differences in the proportions of small and large of the CC group with those groups that consumed sucrose at some periods of their life were found. In both comparisons, sucrose consumption affected in some way the development of large adipocytes, resulting in a significant decrease in the relative percentage of frequency of small adipocytes (CC: 32.6%, CS: 16.1%, SC: 15.4%, and SS: 17.3%) and an increase in large adipocytes (CC: 29.5%, CS: 54.0%, SC: 52.6%, and SS: 51.0%). The highest percentages in the adipocyte CSA were located between intervals (bin 200) of 1,200–1,800 μm^2^ (overall) in all groups (CC: 37.7%, CS: 29.8%, SC: 31.9%, and SS: 32.5%) without differences between them (Figure 7).

These differences between the proportion of small and large adipocytes were observed when drawing with different colors the classification of the CSA adipocyte in the photomicrographs taken at ×10, presenting a high proportion of small adipocytes in the CC group (Figure 6A) compared to the groups that consumed sucrose at some stage of their life, since the CS (Figure 6B), SC (Figure 6C), and SS (Figure 6D) groups presented a greater number of large than small adipocytes.

The adipocytes of the CC group were observed to be proportionally amorphous, asymmetric, and smaller (Figure 6A) compared to those of the CS, SC, and SS groups (Figures 6B–D), which were observed to be more rounded, symmetric, and larger.

## Discussion

Excessive sugar consumption during gestation is positively associated with maternal weight gain at the end of this period (Renault et al., 2015). Findings of the present study confirm that mothers who consumed sucrose throughout gestation increased the consumption of water with sucrose and in consequence the total carbohydrates amount, resulting in a significant increase in the weight at the end of this period. This is because carbohydrate consumption consequently leads to an accumulation of triglycerides in different WAT deposits, which increase their size (hypertrophy) (Morigny et al., 2021). At birth, the consumption of sucrose increased slightly (not significant data) the body length of the offspring. This is likely associated with the glycemic index, which is related to neonatal central adiposity measured by the ratio waist–length in humans (Horan et al., 2014). For its part, birth weight was unaffected by sucrose consumption, as it has been reported in other studies with a moderate maternal nutritional excess (Petherick et al., 2014).

Both in pregnant mothers and adult male groups, there were no differences along the growth trajectory. But a positive relationship was found between the increase in the body weight and weight gain at the end of treatments because the postnatal diet and its interaction with maternal diet, as it had been reported in mice (Mao et al., 2018). Highlighting that in our model, the weight increase begins after the 3rd week of sucrose consumption. This effect on the body weight has been previously described with high carbohydrates diets (Adekunbi et al., 2016; Mao et al., 2018; De León-Ramírez et al., 2021), indicating that the consumption of these sugars (regardless of whether it is mono or disaccharide) is associated with the regulation of solid food intake to adapt energy provided by the increase in the consumption of sugar water (Sheludiakova et al., 2012; Cervantes-Rodríguez et al., 2014).

Respect to the protein consumption, despite the fact that their caloric intake was reduced, it did not imply a deficiency in the consumption of this nutrient, since both in pregnant females that consumed sucrose (S, 18.5%), as in the adult offspring that postnatally consumed it (CS, 19.4%; SS, 19.2%) and in those males control that came from mothers that consumed during pregnancy and breastfeeding (SC, 23%). This intake ranged from 15% to 20% of total caloric intake, which is considered within the optimal range, even between 20% and 25%, which is desirable for these animals (Moro et al., 2021).

We did not observe changes in the number of seminiferous tubules counted, since, as already described, the rat testicle is composed of approximately 20 seminiferous tubules (Clermont and Huckins, 1961), with a spatial distribution that depends on its connection with the rete testis (Nakata et al., 2015). Also, because the complete development of the number of these tubules occurs during the gestational stage, changes in this variable have been reported when interventions by mutagenic, teratogenic, or carcinogenic agents have been carried out at this stage (Tirpák et al., 2021).

Blood–testicular barrier (HTB) maintains the balance in the testicular environment, promoting a healthy and complete development of cell types in these organs (Banks et al., 1999). However, it has been reported that high concentrations of sugar ingested in postnatal stages (Mao et al., 2018; De León-Ramírez et al., 2021) can modify apoptotic processes in the germ and Sertoli cells (Mao et al., 2018), as well as the CSA, epithelial area (germinative, luminal, interstitial), and testicular weight (De León-Ramírez et al., 2021). Probably due to significant increases in the leptin levels that could cross HTB and modify the communication with testicular triglycerides or with important hormones such as testosterone (Mao et al., 2018; De León-Ramírez et al., 2021). In the same context, Despite not finding structural changes in the morphology of the main cell types intra (Sertoli cells, spermatogonia, spermatocytes, spermatids, and spermatozoa) and extra tubular (Leydig cells), we observed a high proportion of macrophages and interstitial mast cells in groups that consumed sucrose maternal and postnatally, which can be related to a pro-inflammatory status with the possibility of affecting testosterone synthesis and its consequent metabolic and cellular interactions (Harris et al., 2016; Ye et al., 2021), including its communication with WAT deposits like the perigonadal.

WAT had hypertrophy and hyperplasia, and the balance between these two mechanisms is an important factor determining the ultimate outcome in lipid storage homeostasis. For this reason, the determination of the size and number of adipocytes in different WAT deposits has focused on finding new and better strategies for their evaluation and understanding of the involved metabolic dynamics (Ibáñez et al., 2018).

Although in our study a Gaussian-type distribution was determined for adipocytes size, it also present characteristics that could be considered a gamma distribution, which has been used to evaluate the size adipocytes distribution from offspring due to the effect of obesogenic maternal diet (Ibáñez et al., 2018).

As it has been reported for the WAT, an increase in the content of triglycerides may be associated with hypertrophy and/or hyperplasia of adipocytes of the PAT (Morigny et al., 2021). We found that both processes were triggered and increased by the high postweaning sucrose intake and programmed by the same diet during pregnancy and lactation. A decrease in the relative percentage of small adipocytes was observed in adulthood, leading to a significant increase in the number and the percentage of large adipocytes in the SC and SS groups. Although maternal diet promotes changes in the WAT physiology, metabolism, and size in adult offspring (Cervantes-Rodríguez et al., 2014; Ibáñez et al., 2018), our data support the theory of fetal programming (Barker, 1990) in periods of development, such as pregnancy and breastfeeding (Renault et al., 2015; Ibáñez et al., 2018; Corona-Quintanilla et al., Online ahead of print), which is probably due to the WAT ontogenesis approximately begins at embryonic day 13.5 (Berry et al., 2016) from the dermomyotome of the mesoderm, and the origin of hypertrophic adipocytes has been speculated since the fetal period and continues after birth (Nielsen et al., 2016; Sebo and Rodeheffer, 2019).

Despite weight gain in PAT and probable metabolic dysfunctions (increased triglycerides storage and adipogenesis) in adipocytes during pregnancy and breastfeeding have been reported, which can lead to the development of hypertrophy and hyperplasia in adulthood, the consumption of a high-sugar diet from early childhood to adulthood promotes the development of these pathologies in adulthood. Although this programming was due to the high consumption of carbohydrates in the maternal diet, the chronic consumption during postnatal life (second hit) of additional simple carbohydrates (such as sucrose) can increase the weight gain in the WAT of the perigonadal deposit and changes the size distribution of its adipocytes. This has been reported in the retroperitoneal adipose tissue of male offspring of rats with maternal obesity on high-fat diets (Ibáñez et al., 2018) and in models with maternal protein deficiency and sucrose consumption (second hit) in postnatal life (Cervantes-Rodríguez et al., 2014).

Conventional approaches often consider variation in the distribution of small and large adipocytes as outliers, rather than functionally significant data with important physiological implications (Ibáñez et al., 2018).

The congruence between results of our work and those of other colleagues (Cervantes-Rodríguez et al., 2014; Ibáñez et al., 2018) could explain the metabolic heterogeneity between obese and non-obese individuals (McLaughlin et al., 2014; Ibáñez et al., 2018), since none of our individuals showed an increase in the body weight considered within the range of obesity, but there is an increase in the body weight of the TAP and in the proportion and size of large adipocytes in this WAT deposit, which is the result of a significant increase in the accumulation of lipids in mature adipocytes (Cervantes-Rodríguez et al., 2014, Cinti et al., 2019), leptin increase (Cervantes-Rodríguez et al., 2014; Ibáñez et al., 2018), and changes in adipogenesis process (Cinti et al., 2019). Further studies are needed to elucidate the development of changes in this process. This together provides a better understanding of the metabolic implications of the variation in WAT.

In addition, hypertrophy of adipocytes from different fatty deposits, including the perigonadal is associated with an increase in the leptin expression, leptin mRNA levels, presence of macrophages, and high levels of IL-6 and TNFα (McLaughlin et al., 2014), as well as a decrease in the insulin levels (Guo et al., 2004).

This increase in the leptin levels (associated with perigonadal adipocyte hypertrophy) can decrease testicular testosterone synthesis (Tena-Sempere et al., 1999; Rato et al., 2013), since there are leptin receptors in Leydig cells, and it has been proven that this protein can cross the HTB and binds to its receptor (Banks et al., 1999), negatively affecting different processes, such as spermatogenesis (germ cell apoptosis), Sertoli cell metabolism (Banks et al., 1999; Mao et al., 2018; Sengupta et al., 2019), and steroidogenesis (by feedback *via* the hypothalamus and pituitary gland), also due to a decrease in insulin associated with high levels of leptin after high sugar diets (Rato et al., 2013; Sengupta et al., 2019). Furthermore, at the level of sperm maturation in the epididymis, it subsequently decreases sperm quality (De León-Ramírez et al., 2021), and even neuroendocrine ones, such as the displacement of adequate sexual behavior (Shulman and Spritzer, 2014). However, more studies are needed to corroborate these hypotheses.

This decrease in the synthesis of testosterone can be accentuated by the pro-inflammatory environment in the interstitial space of the testis, characterized by an increase in the presence of macrophages (Harris et al., 2016), which could be observed in our results.

During the excessive consumption of nutrients, the expansion capacity of WAT reaches its limit and there is a strong association between the size and death of adipocytes (Strissel et al., 2007; Morigny et al., 2021). This triggers changes in the immune cells surrounding hypertrophic WAT adipocytes, increasing the synthesis of pro-inflammatory macrophages and their production of cytokines such as IL-6, TNFα, and oncostatin M (Crewe et al., 2017; Cinti et al., 2019; Morigny et al., 2021). This chronically leads to leptin increase and impaired insulin signaling in adipocytes, increased inflammation, and prolonged worsening of adipose tissue dysfunction (Crewe et al., 2017; Cinti et al., 2019). Although adipose tissue inflammation has detrimental effects, it is possible adaptive and homeostatic roles for pro-inflammatory signaling in WAT expansion and function (Cinti et al., 2019). Thus, the associated metabolic damage (insulin resistance, rise in lipid accumulation, synthesis and expression of leptin, and pro-inflammatory status) (Cervantes-Rodríguez et al., 2014; Ibáñez et al., 2018) can be significantly increased, resulting in alterations in the size of the adipocytes and a considerable increase in the weight of the PAT. Also, it can affect the testosterone synthesis in the testis, promoting actions of leptin in Leydig cells (Banks et al., 1999; Guo et al., 2004).

The results of this study show that the high consumption of simple carbohydrates such as sucrose during pregnancy promotes the establishment of hyperplasia and hypertrophy in WAT deposits such as perigonadal in the male offspring. The development of these processes significantly depends on the amount and type of carbohydrates ingested or not throughout postnatal life. This invites us to pay special attention to the unnecessary consumption of simple carbohydrates during the stages of pregnancy, lactation, early childhood, adolescence, and adulthood.

## Data Availability

The raw data supporting the conclusion of this article will be made available by the authors, without undue reservation.

## Abbreviations

BMI, body mass index; BW, body weight; C, control; CC, control-control; CS, control-sucrose; CSA, cross-sectional area; HTB, blood–testicular barrier; MD, maternal diet; PAT, perigonadal adipose tissue; PNd, postnatal days; PND: postnatal diet; S, sucrose; SC, sucrose-control; SS, sucrose-sucrose; WAT, white adipose tissue.
