# Corrigendum: Maternal and offspring sugar consumption increases perigonadal adipose tissue hypertrophy and negatively affects the testis histological organization in adult rats

**DOI:** 10.3389/fcell.2022.1092235

**Published:** 2022-11-17

**Authors:** Gabriela Córdoba-Sosa, Leticia Nicolás-Toledo, Margarita Cervantes-Rodríguez, Nicté Xelhuantzi-Arreguin, María de Lourdes Arteaga-Castañeda, Elena Zambrano, Estela Cuevas-Romero, Jorge Rodríguez-Antolín

**Affiliations:** ^1^ Doctorado en Ciencias Biológicas, Universidad Autónoma de Tlaxcala, Tlaxcala, Mexico; ^2^ Centro Tlaxcala de Biología de la Conducta, Universidad Autónoma de Tlaxcala, Tlaxcala, Mexico; ^3^ Licenciatura en Nutrición, Facultad de Ciencias de la Salud, Universidad Autónoma de Tlaxcala, Tlaxcala, Mexico; ^4^ Licenciatura en Medicina, Universidad Popular del Estado de Tlaxcala, Tlaxcala, Mexico; ^5^ Licenciatura en Enfermería y Obstetricia, Facultad de Ciencias de la Salud, Universidad Autónoma de Tlaxcala, Tlaxcala, Mexico; ^6^ Departamento de Biología Reproductiva, Instituto Nacional de Ciencias Médicas y Nutrición Salvador Zubirán, Ciudad de México, Mexico

**Keywords:** maternal programming, perigonadal adipose tissue, hypertrophy, testis, high sugar intake

In the published article, the author names in the **Citation** were incorrectly written. The citation should read:

“Córdoba-Sosa G, Nicolás-Toledo L, Cervantes-Rodríguez M, Xelhuantzi-Arreguin N, Arteaga-Castañeda ML, Zambrano E, Cuevas-Romero E and Rodríguez-Antolín J (2022) Maternal and Offspring Sugar Consumption Increases Perigonadal Adipose Tissue Hypertrophy and Negatively Affects the Testis Histological Organization in Adult Rats. Front. Cell Dev. Biol. 10:893099. doi: 10.3389/fcell.2022.893099

In the published article, an **Author name** was incorrectly written as “Xelhuantzi-Arreguín Nicté”. The correct spelling is “Xelhuantzi Arreguin Nicté.”

The authors apologize for this error and state that this does not change the scientific conclusions of the article in any way. The original article has been updated.

